# Patterns and predictors of smoking relapse among inpatient smoking intervention participants: a 1-year follow-up study in Korea

**DOI:** 10.4178/epih.e2021043

**Published:** 2021-06-09

**Authors:** Seung Eun Lee, Chul-Woung Kim, Hyo-Bin Im, Myungwha Jang

**Affiliations:** 1 Center for Tobacco Control Research, Chungnam National University, Daejeon, Korea; 2 Daejeon‧Sejong Tobacco Control Center, Daejeon, Korea; 3 Department of Preventive Medicine and Public Health, Chungnam National University College of Medicine, Daejeon, Korea; 4 Institute of Health and Environment, Seoul National University, Seoul, Korea

**Keywords:** Smoking cessation, Nicotine, Maintenance, Survival analysis

## Abstract

**OBJECTIVES:**

This study aimed to identify relapse patterns in smokers who participated in an inpatient treatment program and to investigate factors related to relapse.

**METHODS:**

The participants comprised 463 smokers who participated in an inpatient treatment operated by the Daejeon Tobacco Control Center from 2015 to 2018. Participants received high-intensity smoking cessation intervention for 5 consecutive days, including pharmacotherapy and behavioral support, and continued with follow-up for 1 year to determine whether they maintained smoking cessation after discharge from inpatient treatment. Kaplan-Meier and Cox proportional hazard models were used in the analysis.

**RESULTS:**

Participants’ relapse rate within 1 year was 72.8%, and 59.8% of participants smoked again within 6 months after participation. A higher number of counseling sessions was significantly associated with a lower risk of relapse (hazard ratio [HR], 0.23; 95% confidence interval [CI], 0.17 to 0.32 for ≥9 vs. ≤5 counseling sessions). Conversely, higher relapse rates were significantly associated with the use of nicotine replacement therapy (NRT) (HR, 1.91; 95% CI, 1.43 to 2.55 for use vs. no use), and higher levels of baseline expired carbon monoxide (CO) (HR, 1.58; 95% CI, 1.21 to 2.06 for expired CO concentrations of 10-19 ppm vs. expired CO concentrations <10 ppm).

**CONCLUSIONS:**

High-intensity smoking cessation interventions in hospital settings can be effective for smoking cessation in smokers with high nicotine dependence. In addition, the results suggest that for quitters to maintain long-term abstinence, they should receive regular follow-up counseling for 1 year after completing a high-intensity smoking cessation intervention.

## INTRODUCTION

Smokers quit and relapse several times before they eventually achieve sustained abstinence [1]. Relapse of smoking after attempting to quit most frequently occurs within the first few weeks [2]. Approximately 75% of smokers experience relapse within 6 months [3]. However, the likelihood of relapse decreases after 6 months to 12 months of abstinence, as 60% to 70% of smokers abstaining for at least 6 months maintain smoking cessation for at least 8 years [1,3,4]. In this respect, 6-month or 12-month abstinence rates are proxies for life-long abstinence [4]. Six-month and 12-month abstinence rates vary according to the type of intervention. The abstinence rate in untreated smokers was found to be between 3% and 5% at 6 months to 12 months, whereas it was approximately 7% in those who received brief advice from a health professional and between 10% and 12% in those who received individual behavioral counseling [5]. Interventions that combine pharmacotherapy, including nicotine replacement therapy (NRT), varenicline and bupropion, and behavioral support (e.g., counseling) have 1-year abstinence rates between 20% and 30% [6-8].

It is difficult to conduct follow-up for longer than 1 year; therefore, most previous smoking cessation studies either used crosssectional designs or focused on specific population groups, such as patients with specific diseases [9]. In Korea, longitudinal studies of people who have tried to quit smoking with follow-up continuing beyond 1 year are scarce. A few domestic studies observed relapse patterns over more than 1 year in individuals who had successfully quit smoking for 6 months after participating in public health center-based smoking cessation clinics [10,11]. Domestic and international studies reported that a lower risk of long-term relapse was associated with older age [12], being married [10,13], higher socioeconomic status [6,14], lack of other household smokers [6,13], lower initial nicotine dependence [9], increased abstinence duration [12,15], motivation to change [6], and smoking cessation aids [8,9,11].

In 2015, the Korean Ministry of Health and Welfare (MOHW) established Regional Tobacco Control Centers in 17 metropolitan cities and provinces that operate inpatient treatment programs [16] modeled after the Mayo Clinic inpatient model of nicotine dependence care [17] and modified for Korea. Since inpatient treatment programs provide intense interventions in a hospital setting, high long-term smoking cessation effects are expected. However, to our knowledge, only 2 studies have analyzed the 6-month smoking cessation success rates in Korea following discharge from an inpatient treatment program [18,19]. Furthermore, these studies did not include variables regarding interventions such as NRT, and they only evaluated smoking cessation for 6 months; therefore, they provide no information on longer-term abstinence.

Therefore, this study aimed to identify relapse rate patterns over 1 year in smokers who participated in an inpatient treatment program at the Daejeon Tobacco Control Center (DTCC), one of the Regional Tobacco Control Centers, and to identify factors related to smoking cessation or relapse for 1 year following treatment.

## MATERIALS AND METHODS

### Procedures

Candidate selection criteria for the inpatient treatment program and all procedures related to the DTCC program operation were implemented in accordance with the related MOHW guidelines [16]. The DTCC conducted online and offline publicity to recruit heavy smokers meeting at least 1 of the following criteria: (1) had smoked for more than 20 years, had failed at least 2 attempts to quit, but had high willingness to quit smoking; or (2) continued to smoke despite a diagnosis of a smoking-related disease, such as cancer or stroke. Primary screening was conducted over the phone to ensure that applicants met the target criteria. Secondary screening included an in-person visit to the DTCC, where candidates were interviewed by a counselor. Each participant completed a registration card providing their demographic characteristics, smoking-related characteristics, and motivation for smoking cessation.

On average, 10 participants were admitted to each 5-day, 4-night inpatient intervention at the Clinical Research Center of Chungnam National University Hospital, a DTCC-affiliated hospital. The daily schedule was strictly structured: the main program included psychological counseling to strengthen motivation to quit smoking, wherein each participant received five 2-hour group counseling sessions and two 20-minute to 30-minute individual counseling sessions with clinical psychologists. In addition, health status checks, lectures, stress management, exercise, and diet therapy were provided. The details of the 5-day program have been described in a previous study [19]. After completing inpatient treatment, participants received follow-up counseling for 6 months via telephone or face-to-face counseling. A gift certificate with a value of 50,000 Korean won (KRW; equal to approximately US$45) was provided as an incentive to those who quit smoking for 6 months. Between 2015 and 2018, 59 inpatient treatment sessions were conducted, each with between 4 participants and 17 participants.

### Data source

Data were compiled from each participant’s electronic record in the Integrated Information System for Smoking Cessation Services (https://nosmk.khealth.or.kr/). This study included 535 individuals who completed the DTCC inpatient treatment between September 1, 2015 and December 31, 2018. Seventy-two participants were excluded from the analyses because we could not confirm their smoking cessation status 1 year after discharge from the program, leaving 463 participants for the analyses.

### Outcome definitions

The dependent variable was smoking cessation duration, defined as the time between a quit attempt and relapse, where relapse was defined as smoking again after participating in the inpatient intervention and successfully quitting. To calculate the duration of smoking cessation, participant relapse was evaluated at 2 weeks, 4 weeks, 12 weeks, and 48 weeks after the date of admission. Relapse at 2 weeks and 4 weeks was evaluated by participants’ expired carbon monoxide (CO) concentrations, where measures exceeding 10 ppm were considered a relapse [20]. Urinary cotinine testing was used to assess relapse at 12 weeks and 24 weeks, where a positive result was identified as a relapse [18]. Relapse at 1 year was assessed through self-reporting in telephone interviews, where a “yes” response to, “Are you currently a cigarette smoker?” was defined as relapse [9].

### Other variable definitions

Factors that can potentially affect relapse in heavy smokers were selected based on previous studies. Demographic characteristics included age, gender, and education level [10,12]. Baseline smoking characteristics included age at smoking onset, smoking years, mean number of cigarettes daily, smoking pack-years, expired CO concentration, and nicotine dependence [9,19]. Expired CO concentration was categorized as < 10 ppm, 10-19 ppm, and ≥ 20 ppm [19]. Nicotine dependence was measured using the Korean version of the Fagerstrőm test for Nicotine Dependence (FTND) scale [21] which comprises 6 items and is scored as 0-3 (low), 4-6 (moderate), and 7-10 (high) [22]. Motivational characteristics included the importance of quitting, confidence in quitting, and readiness to quit, measured using the following questions: (1) How important is quitting smoking to you? (2) How confident are you about succeeding at quitting smoking? (3) How prepared are you to quit smoking? Participants were asked to score each item, ranging from 1 (very low) to 10 points (very high) [23,24]. Intervention methods included smoking cessation counseling and the use of pharmacotherapy [6,25]. The total number of counseling sessions was calculated by adding the number of face-to-face and telephone counseling sessions. Pharmacotherapies were classified as NRT (including nicotine patches, lozenges, and gum) and prescription medications (including varenicline or bupropion) [26,27].

### Statistical analysis

Data were analyzed using IBM SPSS version 24.0 (IBM Corp., Armonk, NY, USA). First, univariate analyses such as the chisquare test and t-test were used to identify differences in participant characteristics between those who relapsed and those who maintained abstinence. Second, the Kaplan-Meier method was applied to estimate the relapse rate and determine the relapse risk over time. Third, a Cox proportional hazards regression model controlling for other factors was performed to identify factors related to relapse and to calculate the hazard ratio (HR), and 95% confidence interval (CI). In addition, tolerance and variance inflation factor values were calculated to examine multicollinearity among the multivariate analysis variables. Multicollinearity was not observed for any of the variables, so all of the variables presented in the results were included in the model.

### Ethics statement

This study was approved by the Institutional Review Board of Chungnam National University (approval No. 201907-SB-104-01). Informed consent was obtained from all participants at the time of their enrollment in the smoking cessation program.

## RESULTS

### Descriptive statistics

The 463 participants’ mean age was 54.5 years, and 90.3% were men. Furthermore, 60.9% of the participants had an expired CO concentration of 10 ppm or higher, and 75.8% had moderate or higher nicotine dependence. Participants received an average of 7.4 smoking cessation counseling sessions; 22.5% of the participants used NRT for smoking cessation, and 2.4% were prescribed smoking cessation medications, such as varenicline or bupropion. The majority (n = 337 of 463; 72.8%) of participants relapsed within 1 year, whereas 126 (27.2%) maintained abstinence for the full year (Table 1).

### Relapse patterns over time

**Figure 1.**
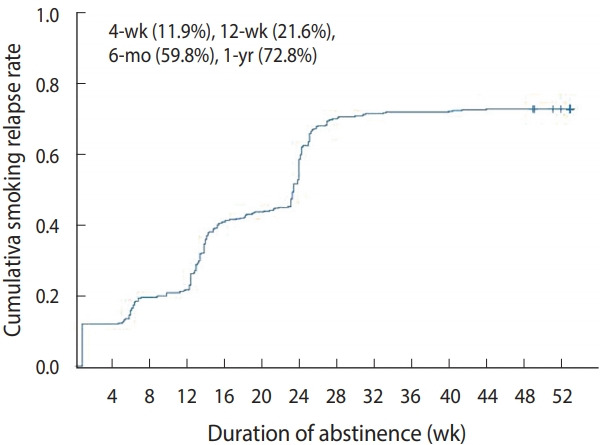
Patterns of relapse over time.

**Figure 2.**
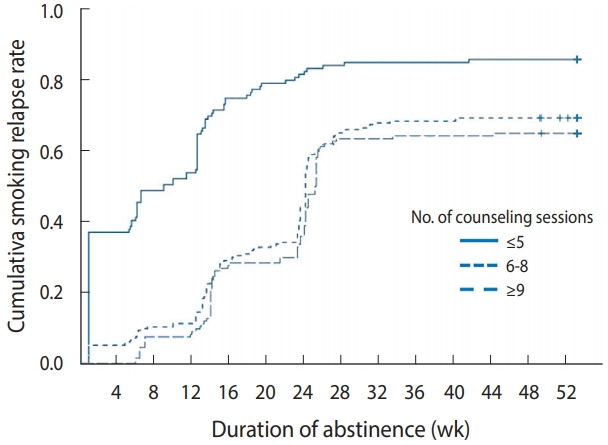
Patterns of relapse over time according to the number of counseling sessions.

Among the 337 participants who relapsed, the greatest proportion of relapses occurred between 13 weeks and 24 weeks after quitting (52.5%), and 82.2% of all relapses occurred within 24 weeks. By the number of counseling sessions, relapse within 4 weeks was most common (43.1%) among those who received ≤ 5 counseling sessions, while relapse within 13-24 weeks was the most common among those who received ≥ 6 counseling sessions (Table 2). These patterns are shown in the cumulative relapse rate curve, where the rate rapidly increased until the first 6 months (24 weeks) after quitting, with 59.8% of participants who tried to quit relapsing within that period. The cumulative relapse rate then increased relatively slowly for the following 6 months, during which 32.3% of those who maintained abstinence for 6 months relapsed (Figure 1). The marked increase in the slope of the relapse curve until 6 months was relatively consistent among all categories with regard to the number of counseling sessions. However, additional counseling sessions delayed the timing of relapse (Figure 2).

### Factors related to relapse

Table 3 shows the results of the Cox proportional hazards model. Older individuals had a lower risk of relapse, with a risk ratio of 0.98 (95% CI, 0.97 to 1.00). The relapse risk was 32% lower in graduates with university degrees or higher compared to those with a middle school or lower degree (HR, 0.68; 95% CI, 0.49 to 0.95). The relapse risk was 1.58 times higher among those with an expired CO concentration of 10 ppm to 19 ppm than among those with an expired CO concentration of less than 10 ppm (95% CI, 1.21 to 2.06). The number of counseling sessions was significantly associated with relapse in all categories. The risk for relapse was 77% lower among those with ≥ 9 counseling sessions than among those with ≤ 5 counseling sessions (HR, 0.23; 95% CI, 0.17 to 0.32). The relapse risk was 1.91 times higher among those using NRT than among those not using NRT (HR, 1.91; 95% CI, 1.43 to 2.55). Gender, age at smoking onset, smoking pack-years, nicotine dependence, and the use of anti-smoking medications were not significantly associated with relapse risk.

## DISCUSSION

This was the first study to evaluate the 1-year abstinence rate and relapse timing among smokers who attempted to quit by participating in the national inpatient treatment for smoking cessation operated by the MOHW. The cumulative relapse rate within 1 year was 72.8%, and 27.2% of all participants maintained abstinence for 1 year. These results reflect lower performance compared to the 1-year abstinence rates between 29% and 45% reported from the Mayo Clinic’s inpatient treatment program [17,28], which was used as a benchmark for developing the Korean inpatient treatment model. Since inpatient treatment in Korea is fully sponsored by the government, participation in the program was free. In contrast, participants in the Mayo Clinic’s program had to pay approximately US$2,800 [17,28], making it highly likely that participants with a high income generally participated in the program. The percentage of high school graduates was 83.8% in our inpatient treatment program, but 97.9% in the Mayo Clinic program [28]. Previous studies reported that high socioeconomic status was associated with a high abstinence rate [6,14], and the difference in the abstinence rate may be explained by the gap in socioeconomic status among the participants. Other international studies have reported 1-year abstinence rates between 10% and 12% when individual behavioral counseling was provided [5], and between 20% and 30% when smoking cessation interventions were combined with behavioral support interventions, including multisession group therapy programs or individual counseling sessions, and pharmacological treatment [1,6,7]. Compared with the results of the aforementioned studies, the results of this study suggest that the inpatient treatment program provided both behavioral support and pharmacological treatment, and thus yielded a relatively high abstinence rate although the participants were recruited among heavy smokers.

Our findings showed that the relapse rate rapidly increased up to 6 months after quit attempts, with 59.8% of participants relapsing within the first 6 months. This supports previous studies [6,9,11] reporting that relapse was most likely to occur in the first 6 months following treatment. Although the relapse rate showed a decreasing pattern after 6 months, 32.3% of those who maintained smoking cessation for 6 months relapsed within the following 6 months, which is consistent with previous studies [10,11]. Our results showed that the relapse rate increased rapidly within the first 6 months after quit attempts, and that about one-third of the 6-month abstainers relapsed within the following 6 months. These results suggest that intensive relapse prevention measures should be provided for the first 6 months following inpatient treatment, and regular follow-up should be conducted for at least 1 year to induce long-term smoking cessation. Nohlert et al. [1] described that 1-year follow-up status was a strong predictor of long-term smoking abstinence, suggesting that counselors’ structured long-term contacts might increase the likelihood of exposure to positive reinforcement and skill training. Previous studies have reported that 60% to 70% of 1-year quitters maintained long-term abstinence for more than 5 years [3,4].

We found that the risk factors related to relapse within 1-year included age, education level, expired CO concentration, the importance of quitting, confidence in quitting, readiness to quit, number of smoking cessation counseling sessions, and NRT use. Among these factors, the intervention characteristics, such as the number of smoking cessation counseling sessions and NRT use, showed a higher association with relapse risk than socio-demographic characteristics, initial smoking characteristics, and participants’ motivational readiness.

First, a higher number of smoking cessation counseling sessions was found to be associated with a lower relapse rate, which is consistent with the results of previous studies [1,11,19,22,24] showing significant associations between the number of smoking cessation counseling sessions and smoking cessation success rates in many different populations and settings. Lancaster & Stead [5] conducted a meta-analysis of 49 studies on the effects of face-to-face individual counseling and found that 7 of 100 individuals maintained abstinence for at least 6 months when brief advice or self-help materials were provided, and 10-12 of 100 individuals successfully quit smoking when counseling was provided. In addition, 11 of 100 individuals successfully quit smoking with medications such as NRT, whereas 11-16 of 100 individuals successfully quit when counseling was added [5]. Although the optimal intensity of counseling interventions has not been clearly defined, many previous studies have reported that high-intensity programs that included more counseling sessions led to higher cessation rates [1,5,7,8]. The inpatient smoking cessation treatment program described herein provides high-intensity counseling through 5 group counseling and 2 individual counseling sessions during the program period, and continues face-to-face or telephone counseling for 6 months after inpatient treatment completion. Such high-intensity intervention showed a relatively high abstinence rate. Although this study did not analyze the effects of counseling on smoking cessation by counseling type, previous studies found that both telephone counseling and face-to-face counseling were effective for smoking cessation [5,29]; both group interventions and individual interventions were also effective, and there was no significant difference between the 2 counseling methods [30]. Future studies of the smoking abstinence rate by time period according to counseling type will provide information for designing and operating cost-effective inpatient treatment programs.

Only NRT pharmacotherapy was found to be significantly associated with relapse risk, whereas medications such as varenicline or bupropion were not significantly associated with relapse risk. Many clinical trials and population studies have reported that NRT, in the form of nicotine patches, gum, or other products, helped quitters to effectively maintain smoking cessation for 6 months or long-term abstinence for more than 1 year [3,8,12,26,31]. However, this study found that relapse risk was 1.91 times higher in the NRT group than in the non-NRT group. This supports the results of domestic studies [10,11,32], wherein relapse risk was 1.36 times to 2.93 times higher among NRT users than among non-users. However, these results may not indicate that NRT use increased relapse risk, because the NRT use period was very short [32], or the temporal relationship between relapse and NRT use was unclear [10]. Currently, the inpatient treatment program provides NRT, such as gum and patches, during a hospital stay and until 6 months after discharge to those who request them, after the counselor takes the participant’s preference and amount of smoking into account. Considering this process, NRT may be used by participants with high nicotine dependence, thus resulting in a high risk of relapse, which may have contributed to the results [11]. Previous studies have reported that varenicline, bupropion, and NRT increased long-term success [8,26], with varenicline being more effective than NRT [25-27]. However, few participants in this study used varenicline or bupropion, so the effects of those drugs could not be identified. To evaluate the effects of NRT, varenicline, or bupropion on smoking cessation, more sophisticated study designs considering their types and amounts are needed.

Among participants’ characteristics, baseline expired CO concentration was significantly associated with relapse risk; specifically, relapse risk was higher among those with an expired CO concentration of 10 ppm to 19 ppm than among with a concentration less than 10 ppm. This result is similar to the results of previous studies [10,24] in which the failure rate of smoking cessation was higher in individuals with a high expired CO concentration than in those with a low expired CO concentration. CO concentration reflects smoking status and amount of smoking. Since a high expired CO concentration indicates severe nicotine dependence [25], longer-term and more intensive management is needed after inpatient treatment completion. Age at smoking onset, smoking pack-years, and FTND score were not significantly associated with relapse risk in this study. This result is similar to those of previous domestic and international studies [10,18,19, 25,28] reporting that smoking history-related characteristics, such as age at smoking onset, smoking duration, and amount of smoking, were not significantly associated with smoking cessation success for more than 6 months. Other studies reported that a higher FTND score was associated with a higher relapse rate [9,11,22]. However, those studies did not include expired CO concentration as a variable; therefore, the results are not comparable to this study’s results.

This study has the following limitations. First, the 1-year abstinence rate was measured using self-reports and was not biochemically verified. A previous study comparing self-reported and CO-validated 1-year abstinence rates reported that the self-reported abstinence rate was 17.6% higher [6]. Therefore, the actual relapse rate in this study might have been higher than indicated. Second, all variables that may affect relapse were not included in the analytical model used in this study. For example, previous studies [6,14] have found that there was a high probability of relapse within 1 year among those with low socioeconomic status, but a comprehensive range of data measuring socioeconomic status, such as participants’ income level or employment type, could not be obtained in this study. The reason why education level, as a demographic characteristic, was found to be significantly associated with relapse in the results of this study, is thought to be that education level was the only variable reflecting participants’ socioeconomic status. In addition, although previous studies have reported that health status [9,11], baseline duration of abstinence [12,13,15], and other household smokers [6,13] were significantly associated with long-term smoking abstinence for more than 1 year, this study could not obtain related data. Third, since this study involved an inpatient treatment program for heavy smokers operated by a single Tobacco Control Center, the results may not be generalizable to other populations. According to the 2018 Korea National Health and Nutrition Examination Survey, the mean age of current smokers was 46.8 years, 83.2% of current smokers were men, and the mean number of cigarettes smoked daily was 12.9 [33]. The corresponding values (54.5 years, 90.3% men, and 20.5 cigarettes daily, respectively) were different among the participants of the inpatient treatment program. Finally, 27% (n= 72) of the participants with 6-month abstinence after participating in the inpatient treatment program were lost to follow-up, so data on their 1-year abstinence rate were not obtained. However, there were no significant differences in characteristics between those who completed the follow-up and those who were lost to follow-up.

In conclusion, the inpatient treatment program described herein is an intensive nicotine dependence treatment program that provides both individual and group therapies for heavy smokers. Our results showed that about 32% of those who abstained for 6 months eventually relapsed within 1 year. Since inpatient treatment for smoking cessation is a high-cost program for smokers with a higher risk of nicotine addiction, our findings suggest that the follow-up period should be extended to 1 year for inpatient interventions. Providing structured counseling for 1 year may lead to cost-effective outcomes by preventing relapse in smokers with high nicotine dependence and helping them achieve lifelong abstinence.

## Figures and Tables

**Table 1.** Differences in the relapse rate according to participants’ characteristics

Variables			Total (n=463)	Relapsed (n=337)	Abstinent (n=126)	p-value
Demographic characteristics						
	Age (yr)		54.5±10.8	53.4±10.4	55.9±10.6	0.025
		<60	319 (68.9)	241 (71.5)	78 (61.9)	0.047
		≥60	144 (31.1)	96 (28.5)	48 (38.1)	
	Gender					
		Men	418 (90.3)	304 (90.2)	114 (90.5)	0.931
		Women	45 (9.7)	33 (9.8)	12 (9.5)	
	Education level					
		Middle school or less	75 (16.2)	57 (16.9)	18 (14.3)	0.015
		High school	128 (27.6)	104 (30.9)	24 (19.0)	
		University or higher	260 (56.2)	176 (52.2)	84 (66.7)	
Baseline smoking characteristics						
	Age at smoking onset (yr)		20.8±5.3	20.3±4.9	21.2±6.1	0.152
	Total smoking duration (yr)		34.4±10.9	33.7±10.6	35.6±11.1	0.089
	No. of cigarettes daily		20.5±9.2	21.7±9.6	19.2±8.0	0.010
	Pack-years		34.9±18.8	36.27±19.1	34.0±18.6	0.242
	Expired CO concentration (ppm)					
		<10	181 (39.1)	115 (34.1)	66 (52.4)	0.001
		10-19	167 (36.1)	134 (39.8)	33 (26.2)	
		≥20	115 (24.8)	88 (26.1)	27 (21.4)	
	Nicotine dependence (score)					
		0-3 (low)	112 (24.2)	77 (22.8)	35 (27.8)	0.097
		4-6 (moderate)	194 (41.9)	136 (40.4)	58 (46.0)	
		7-10 (high)	157 (33.9)	124 (36.8)	33 (26.2)	
Motivational characteristics (score)						
	Importance of quitting		8.9±1.8	9.0±1.7	8.7±1.9	0.036
	Confidence in quitting		7.2±2.3	7.1±2.5	7.2±2.1	0.735
	Readiness to quit		7.9±2.2	7.9±2.2	7.6±2.2	0.248
Intervention methods						
	No. of counseling sessions		7.4±3.1	6.8±2.9	8.2±3.0	<0.001
		≤5	118 (25.5)	102 (30.3)	16 (12.7)	<0.001
		6-8	212 (45.8)	148 (43.9)	64 (58.8)	
		≥9	133 (28.7)	87 (25.8)	46 (36.5)	
	Use of pharmacotherapies (NRT)					
		No use	359 (77.5)	256 (76.0)	103 (81.7)	0.185
		Use	104 (22.5)	81 (24.0)	23 (18.3)	
	Varenicline or bupropion					
		No use	452 (97.6)	329 (97.6)	123 (97.6)	0.996
		Use	11 (2.4)	8 (2.4)	3 (2.4)	
Smoking status at 1-year follow-up			463 (100)	337 (72.8)	126 (27.2)	

Values are presented as number (%) or mean±standard deviation. NRT, nicotine replacement therapy.

**Table 2.** Relapse by time period after quitting smoking among the inpatient treatment program participants

Variables	Total	No. of counseling sessions		
		≤5	6-8	≥9
Relapse by time period (wk)				
0-4	55 (16.3)	44 (43.1)	11 (7.4)	0 (0.0)
5-12	45 (13.4)	20 (19.6)	13 (8.8)	12 (13.8)
13-24	177 (52.5)	35 (34.3)	95 (64.2)	47 (54.0)
25-48	60 (17.8)	3 (2.9)	29 (19.6)	28 (32.2)
Total	337 (100)	102 (100)	148 (100)	87 (100)

Values are presented as number (%).

**Table 3.** HRs for factors related to relapse within 1 year

Variables	HR (95% CI)
Demographic characteristics	
Age (yr)	0.98 (0.97, 1.00)^*^
Gender	
Men	1.00 (reference)
Women	1.33 (0.86, 2.07)
Education level	
Middle school or less	1.00 (reference)
High school	1.06 (0.75, 1.49)
University or higher	0.68 (0.49, 0.95)^*^
Baseline smoking characteristics	
Age at smoking onset (yr)	0.98 (0.95, 1.01)
Pack-years	1.00 (1.00, 1.01)
Expired CO concentration (ppm)	
<10	1.00 (reference)
10-19	1.58 (1.21, 2.06)^**^
≥20	1.20 (0.88, 1.63)
Nicotine dependence (score)	
0-3	1.00 (reference)
4-6	0.93 (0.69, 1.25)
7-10	0.97 (0.70, 1.35)
Motivational characteristics (score)	
Importance of quitting	1.09 (1.02, 1.17)^*^
Confidence in quitting	0.93 (0.87, 0.99)^*^
Readiness to quit	1.07 (1.00, 1.14)^*^
Intervention methods	
No. of counseling sessions	
≤5	1.00 (0.75, 0.83)
6-8	0.32 (0.24, 0.42)^***^
≥9	0.23 (0.17, 0.32)^***^
NRT	
No use	1.00 (reference)
Use	1.91 (1.43, 2.55)^***^
Varenicline or bupropion	
No use	1.00 (reference)
Use	1.69 (0.80, 3.60)

HR, hazard ratio; CI, confidence interval; NRT, nicotine replacement therapy.

* p<0.05,

** p<0.01,

*** p<0.001.
